# Simultaneous fluorescein sodium and 5-ALA in fluorescence-guided glioma surgery

**DOI:** 10.1007/s00701-015-2401-0

**Published:** 2015-03-28

**Authors:** Michael Schwake, Walter Stummer, Eric Jose Suero Molina, Johannes Wölfer

**Affiliations:** Department of Neurosurgery, University Hospital Münster, Albert-Schweitzer-Campus 1, Gebäude A1, 48149 Münster, Germany

Dear Editor,

Five-aminolevulinic acid (5-ALA)-derived fluorescence is approved for fluorescence-guided resections of malignant gliomas, relying on selective synthesis and accumulation of protoporphyrin IX (PPIX) within malignant glioma cells [5]. However, the first use of fluorescence for brain tumour surgery was in 1948 by G.E. Moore [3] using fluorescein sodium, a strongly fluorescing and non-toxic (apart from rare anaphylaxis [2]) compound. In malignant brain tumours with their inherent blood-brain barrier breakdown, fluorescein is extravasated and might serve to mark tumours.

Today, fluorescein sodium is again under scrutiny [1, 4] using a novel filter system by Zeiss (YELLOW 560) for the microscope. This filter visualises fluorescein and allows good background discrimination. Furthermore, fluorescein can be injected any time and is low in cost. Nevertheless, its use in brain tumour surgery is off-label and thus restricted to clinical studies.

Little is known about the best timing of i.v. fluorescein application before resection. Injecting fluorescein too early might result in unspecific propagation with oedema, whereas acute injections might be useful for detecting abnormally perfused tumour tissue. Levels in the blood will be high, especially with acute injections, leading to fluorescence of all perfused brain tissue. To our knowledge, such time-resolved information on the specificity of fluorescein are not available. Therefore, we conducted a small pilot study using two timing regimes in four patients pre-treated with 5-ALA (20 mg/kg, Gliolan; medac, Wedel, Germany), comparing early (35 min prior to durotomy, as previously described [4]) with acute injections of fluorescein (4 mg/kg, Fluorescein Alcon 10 %; Alcon, Freiburg, Germany). We used a Leica M530 OH6 (Leica Microsystems, Heerbrugg, Switzerland) equipped with a FL400 filter, freely interchangeable with an experimental band pass filter system for detecting fluorescein fluorescence without superimposed background information (Leica FL560) and allowing simultaneous visualisation of both fluorophores.

An example of fluorescein application prior to opening of the dura is given in the Fig. 1. This patient presented with a secondary malignant glioma in the right fronto-temporal region with patchy contrast enhancement. PPIX fluorescence was predominantly noted in one area, shining through the cortex, and was patchier in other areas. Fluorescein was found corresponding to porphyrin fluorescence, but was also visible in the dura, the cerebrospinal fluid (CSF) and weakly throughout the brain. After corticotomy fluorescein was found spreading through the arachnoid space away from the lesion, corresponding to regions of superficial coagulation for extending the corticotomy, along the corticotomy margins and in blood.

**Fig. 1:**
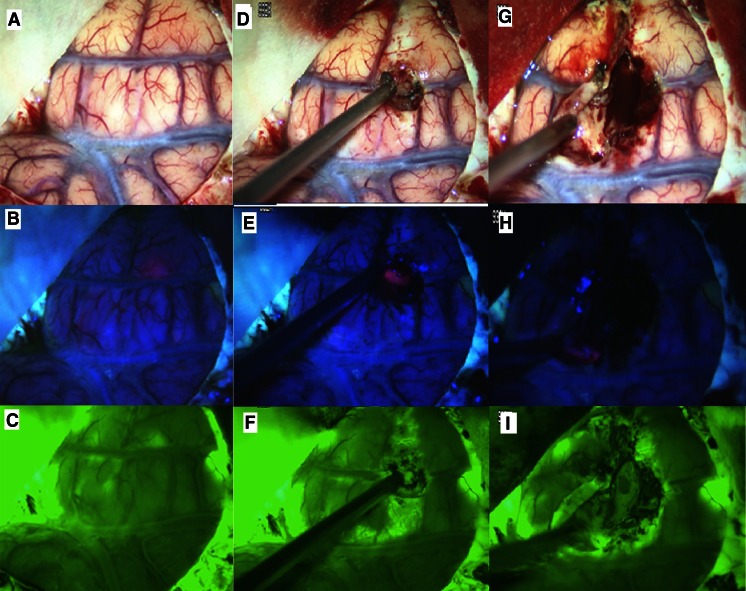
White light (**a**) and PPIX image (**b**) of exposed cortex (proximal perisylvian region) demonstrating patchy PPIX fluorescence shining through the otherwise mostly unremarkable cortex. **c** Fluorescein image after application of fluorescein prior to dura opening, as previously described [4]. There is some indication of fluorescein fluorescence corresponding to PPIX fluorescence. However, *green* fluorescence was also observed in a CSF accumulation at the base of the Sylvian fissure, less strongly throughout the entire cortex and in a small area of cortical damage, presumably imposed during durotomy, is also visible. **d** and **g** White light images after corticotomy; **e** and **h** corresponding PPIX images; **f** and **i** corresponding fluorescein images showing unspecific extravasation in the cortex after coagulation for corticotomy

In a second patient, a patient with recurrent low-grade glioma, in whom fluorescein was given before opening of the dura, fluorescence was found in the brain adjacent to the tumour and in the dura itself. However, fluorescence appeared patchy or missing in the macroscopically detectable tumour. The tumour was not positive for 5-ALA fluorescence. At the end of the resection, after tumour removal had been confirmed by intra-operative ultrasound, diffuse green fluorescence was observed along all resection margins. Biopsies taken from this area were devoid of tumour.

In a third case, a patient with temporal glioblastoma, we attempted acute injection (Supplementary video) to determine whether fluorescein would correspond to porphyrin fluorescence. Fluorescein was visible in normally perfused brain. In the tumour cavity, fluorescein was in fact extravasated in the region of gross and necrotic tumour, but was not or vaguely visible in the marginal tumour. Blue light illumination for PPIX showed the adjacent white matter to carry strong red PPIX fluorescence, signifying tumour. We had a similar experience with a final patient after debulking of the main tumour mass and acute injection.

Overall, we observed no clear value of fluorescein in our small study, which we closed prematurely. Clearly, further work elucidating optimal timing and dosing of fluorescein is warranted.

## Electronic supplementary material

Below is the link to the electronic supplementary material.

Supplementary video Acute injection of fluorescein sodium with the aim of detecting abnormally perfused tumour tissue (as detected by co-administered ALA-induced PPIX fluorescence), relying on possible differential accumulation in the contrast-enhancing tumour positions. Fluorescein was extravasated markedly in the necrotic part of the tumour but only minimally in the tumour surrounding necrosis (verified by biopsy), which was clearly PPIX positive. (M4V 28035 kb)
